# Effect of Dual Modification on the Spectroscopic, Calorimetric, Viscosimetric and Morphological Characteristics of Corn Starch

**DOI:** 10.3390/polym11020333

**Published:** 2019-02-14

**Authors:** Ulin Antobelli Basilio-Cortés, Leopoldo González-Cruz, Gonzalo Velazquez, Gerardo Teniente-Martínez, Carlos Alberto Gómez-Aldapa, Javier Castro-Rosas, Aurea Bernardino-Nicanor

**Affiliations:** 1 Tecnológico Nacional de México-Celaya, Antonio García Cubas Pte #600 esq., Av. Tecnológico, A. P. 57, Celaya 38010, Guanajuato, Mexico; antobeli10@hotmail.com (U.A.B.-C.); leopoldo.gonzalez@itcelaya.edu.mx (L.G.-C.); gera_tm@hotmail.com (G.T.-M.); 2 Instituto Politécnico Nacional, CICATA-IPN unidad Querétaro. Cerro blanco 141, colinas del cimatario, Santiago de Querétaro 76090, Querétaro, Mexico; gvelazquezd@ipn.mx; 3 Área Académica de Química, Instituto de Ciencias Básicas e Ingeniería, Ciudad del Conocimiento, Universidad Autónoma del Estado de Hidalgo, Carretera Pachuca-Tulancingo Km. 4.5, Mineral de la Reforma 42183, Hidalgo, Mexico; cgomeza@uaeh.edu.mx (C.A.G.-A.); jcastro@uaeh.edu.mx (J.C.-R.)

**Keywords:** acid hydrolysis, corn starch, dual modification, succinylation

## Abstract

The effect of dual modification of corn starch, including hydrolysis and succinylation, were evaluated through peak viscosity (PV) analysis, differential scanning calorimetry (DSC), scanning electron microscopy (SEM), Fourier transform infrared spectroscopy (FTIR) and Raman spectroscopy. This dual modification was shown to increase the reaction efficiency (RE) and degree substitution (DS) compared with starches that were not subjected to acid hydrolysis pretreatment with a 44% and 45% increase respectively. After acid hydrolysis pretreatment, the surface of the corn starch granules exhibited exo-erosion and whitish points due to the accumulation of succinyl groups. The peak viscosity was reduced significantly with the acid hydrolysis pretreatment (between 3 and 3.5-fold decrease), which decreased the pasting temperature and peak time to 20 °C and 100 s respectively. In addition, the dual modification of corn starch altered certain thermal properties, including a reduction in the enthalpy of gelatinization (ΔH) and a higher range of gelatinization (around 6 °C), which may effectively improve industrial applications. Modifications on the FTIR spectra indicated that the dual modification affected the starch crystallinity, while the Raman spectra revealed that the dual modification disrupted the short-range molecular order in the starch. Rearrangement and molecular destabilization of the starch components promoted their granular amphiphilic properties.

## 1. Introduction

Starch is a polymer that is synthesized in the form of a granule and used as a gelling agent, stabilizer, thickener or emulsifier in the pharmaceutical, food, textile and packaging industries and for bioethanol production [1,2,3]. Corn starch is one of the most well-studied starches due to its low cost, ease of obtaining and availability; however, corn starch has several disadvantages, such as its large particle size, strong polar surface, high cohesion energy and high soft temperature, generating in starch dispersion, which limits its use in the new product development area [4,5]. However, it has been observed that modifications to the granular structure of corn starch promotes other physicochemical and functional properties [6]. Modifying corn starch by chemical or physical methods would aid in overcoming its undesirable characteristics in order to control its chemical-physical properties, in particular in terms of solubility and gelatinization time.

Acid hydrolysis is one of the commonly used methods for the depolymerization of starch granules to improve certain physicochemical properties, modifying the cooking and gelatinization temperatures that permit obtaining gel of higher strength [7]; on the other hand, starches with an acid treatment show higher solubilities and in long term cold storage shows the higher propensity for retrogradation. The acid hydrolysis pretreatment of the starch, increases the hydroxyl groups, which favors esterification with a functional group (acetyl, phosphate or succinate) [8,9]. It has been observed that the depolymerization of the starch before the succinylation treatment by acid hydrolysis, pyrodextrinization or enzyme hydrolysis, modifies the degree of substitution (DS), reaction efficiency (RE) and the amphiphilic characteristics of the starch [10,11]. On the other hand, the derivatization of starch by introducing succynil groups along the polymeric backbone tends to stabilize starch by preventing or curtailing starch retrogradation and both the solubility and viscosity of the starch are modified along with an increasing degree of substitution. In turn, these modifications result in good gel-forming and emulsifying properties and the capability for film formation [12].

For this reason, dual modified starches are used for the encapsulation of food ingredients, however, such modifications has been evaluated by means of chromatographic methods [13]; but, these techniques are destructive and can consume substantial amounts of resources. For this reason, spectroscopic techniques, such as Fourier transform infrared (FTIR) and Raman (FT-Raman) have been used to analyze modifications of the starch based on various treatments, ultrasonication, heat-moisture treatment and type of extraction [14,15,16].

Several studies have been reported that show that the succinylation process improves starch characteristics for food applications, nevertheless, for improvement of the starch characteristics, several changes on the modification conditions have been tested e.g., temperature, pH, time and starch concentration, with a combined method used in all cases [17,18,19]. However, it has not been reported if dual modification of corn starch is better than a single modification for improving their physical and chemical characteristics.

Few studies have reported the use of FT-Raman and FTIR spectroscopy as tools in the study of the structural changes of the polymeric components that take place in starch granules as a function of hydrolysis and esterification. The goal of this study was to evaluate if the dual modification of corn starch generated the highest difference in their physical and chemical characteristics than a single modification, for future applications as wall material for food encapsulation. For this reason, the present study employed both Raman and FTIR spectroscopy in addition to microscopy and analyses of peak viscosity, thermal properties, and hydrophobicity to investigate the changes occurring in the starch structure and the characteristics resulting from a dual modification (acid hydrolysis and succinylation).

## 2. Materials and Methods

### 2.1. Materials

**Figure 1:**
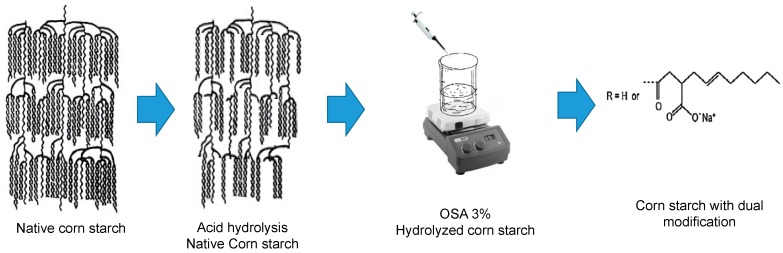
Schematical representation of the dual corn starch modification.

Native corn starch (CS) was purchased from the industrial corn company (IMSA, S.A. de C.V., Mexico). Hydrochloric acid (37%; J. T. Baker, Phillipsburg, NJ, USA) and 2-octen-1-yl succinic anhydride (97% purity, Sigma-Aldrich Co., St. Louis, MO, USA) were used for the dual starch modification as is shown in Figure 1.

### 2.2. Acid Hydrolysis Pretreatment

A total of 100 g of corn starch was suspended in a solution of hydrochloric acid at 5% (300 mL) for approximately 5 h at room temperature. Subsequently, the solution was neutralized with NaOH solution (20%). The neutralized solution was washed three times with distilled water, centrifuged at 6000 rpm for 12 min (Hermle Labortechnik centrifuge, model Z300K, Wehingen, Germany), the supernatant was discarded and the pellet was dried in a forced convection oven (Lab-Line, Ambi-Low Chamber, Livonia, MI, USA) at 40 °C. The dried starch was ground and sieved using a 250 μm mesh. The dried samples were stored for further analysis [20].

### 2.3. Succinylation Treatment

A 40% (*w*/*w*) starch slurry was prepared in distilled water for both starches (native corn starch and acid corn starch), and the pH was adjusted to 8.5 using an NaOH solution (3%). Succinic anhydride (3% *w*/*w* based on the dry weight of starches) was gently added to the starch slurry, maintaining the pH between 8.5 and 9. After the complete addition of the succinic anhydride, the slurry was stirred for 6 h, after which the reaction was terminated by neutralizing the slurry to pH 7 with an HCl solution (0.5 M). Finally, the slurry was dried in a forced convection oven [21].

### 2.4. Degree of Substitution

Samples of octenyl succinate starch (5.0 g) (with or without acid hydrolysis pretreatment) were suspended in water (50 mL). The starch solutions were heated to 50 °C in a water bath and then cooled to room temperature. Next, 25 mL of NaOH solution (0.5 N) was added to each starch solution with continuous stirring. The solutions were covered and left for 24 h with occasional stirring and were subsequently titrated with an HCl solution (0.5 N) using phenolphthalein as an indicator. Native corn starch was used as the blank. The percent succinyl content and degree of substitution (DS) were calculated using equations 1 and 2 as follows [21]:

(1) $$\%\;\mathrm{Succinyl}=\frac{\left(\mathrm{Blank}\;\mathrm{titre}-\mathrm{Sample}\;\mathrm{titre}\right)\times 0.1\times \;\mathrm{normality}\;\mathrm{of}\;\mathrm{acid}\;\times 100}{\mathrm{Weigh}\;\mathrm{of}\;\mathrm{sample}\;\left(g\right)}$$

(2) $$\mathrm{DS}=\frac{162\times \left(\%\;\mathrm{succinyl}/100\times \right)}{100-\left(99/100\times \;\%\;\mathrm{Succinyl}\right)}$$

The reaction efficiency (RE) was calculated using equation 3 as follow:

(3) $$\mathrm{RE}=\frac{\mathrm{Actual}\;\mathrm{DS}}{\mathrm{Theoretical}\;\mathrm{DS}}\times 100$$

The theoretical DS was calculated assuming that all of the added anhydride reacted with starch to form the ester derivative.

### 2.5. Physicochemical Characterization of the Starches

#### 2.5.1. Peak Viscosity (PV)

Peak viscosity was measured using a Rapid Visco Analyzer (3C, Newport Scientific Pty. Ltd., Sydney, Australia). Each sample (2.5 g dry basis) was weighed, and distilled water was added to reach a total weight of 28 g of suspension. The mixture was placed in the analyzer with stirring at 960 rpm for 10 s before being heated from 25 to 50 °C and then held at this temperature for 1 min. Subsequently, the sample was heated from 50 to 92 °C at 5.6 °C/min under 160 rpm shearing conditions and maintained at 92 °C for 5 min. Next, the sample was cooled to 50 °C at the same rate with the same shearing conditions, and then the temperature was maintained at 50 °C for 2 min [22]. Each sample was analyzed in triplicate.

#### 2.5.2. Thermal Properties of the Starches

The thermal properties of the starches were determined using a differential scanning calorimeter (DSC) (DSC-822E; Mettler Toledo AG, Analytical, Schwerzenbach, Switzerland) to evaluate the starch gelatinization and retrogradation. Starch (4 mg) was weighed directly into aluminum pans, hydrated with 16 mL of distilled water, hermetically sealed and equilibrated for 2 h at room temperature prior to the test. The scanning temperature range and heating rate were 25 to 95 °C and 5 °C/min, respectively. An empty pan of the same size was used as a reference. The enthalpy of gelatinization (Δ*H*), gelatinization onset temperature (*T*_o_), gelatinization peak temperature (*T*_p_), and gelatinization conclusion temperature (*T*_c_) were measured from the endotherm in the DSC thermograms. Each sample was analyzed in triplicate.

#### 2.5.3. Scanning Electron Microscopy (SEM)

Samples of native corn starch and octenyl succinate-treated starches (with or without acid hydrolysis pretreatment) were examined using a JEOL scanning electron microscope (model JEOL JSM-6300 Akishima, Tokyo, Japan) fitted with a Kevex Si(Li) X-ray detector. The analyses were performed under vacuum conditions at an accelerating voltage of 15 kV. The samples were mounted on double-sided carbon tape and covered with approximately 10 nm of gold using a Denton sputter coater.

#### 2.5.4. FTIR Spectroscopy

The FTIR spectra of modified corn starch were acquired with a Perkin Elmer FTIR spectrophotometer (Perkin Elmer, Inc., Waltham, MA, USA) using potassium bromide (KBr) discs prepared from powdered samples mixed with dry KBr. The spectra were recorded (16 scans) in transmittance mode in the range of 4000 to 400 cm^−1^.

#### 2.5.5. Raman Spectroscopy

The Raman measurements were obtained on a Perkin–Elmer (Perkin Elmer, Inc., Waltham, MA, USA) 2000R NIR FT-Raman Spectrometer equipped with an Nd:YAG laser emitting at a wavelength of 1064 nm and an InGaAs detector. For the analysis, 180° backscattering refractive geometry was used. The spectrometer was run using Perkin–Elmer Spectrum software (version 3.02.00 [2000]). Spectral data were obtained at a wavenumber resolution of 4 cm^−1^ at a nominal laser power of 500 mW. For each spectrum, 20 scans were accumulated to ensure an acceptable signal-to-noise ratio. All Raman spectra were collected at room temperature.

### 2.6. Statistical Analysis

Quantitative data were expressed as the mean ± standard deviation. Analysis of variance (ANOVA) was performed followed by Tukey’s test with a confidence interval of 95% (*p* ≤ 0.05). Using Origin software v. 8.0 (Origin Inc. Northampton, MA, USA) was employed for the data analysis and all assays were performed in triplicate.

## 3. Results and Discussion

### 3.1. Effect of Dual Modification on the Degree of Substitution

The starches that were subjected to the dual modification exhibited a higher RE compared with the starches without hydrolysis pretreatment (75.90% and 52.60%, respectively) and consequently, a higher DS. The acid hydrolysis pretreatment likely generated more available hydroxyl groups in the starch, which enhanced the succinylation process. However, it was previously observed that the substitution by succinyl groups is not uniform in all starch granules due to the compact structure of the starch, for this reason, the OSA groups appear to primarily esterify the glucose at the starch surface [23,24].

The DS in the starches with dual modification was higher compared with the starches without acid hydrolysis pretreatment (2.086% and 1.43%, respectively). The conditions used in the acid hydrolysis pretreatment resulted in the corn starch granule destabilization and exhibited increased depolymerization increasing polymerization, due to the primary attack of the acid on the amorphous region of the starch granule, where hydroxonium ions hydrolised the glycosidic linkages, consequently a decline in molecular weight of chains was observed [7]. These modifications in the starch structure apparently increased the esterification process of the glucose molecules by the succinyl groups. The acid hydrolysis pretreatment in the dual modification increased the esterification efficiency up to 64%, maintaining the morphology and structure of the starch granule.

According to an FDA report, for the modification of starch with OSA, the highest level permitted is around 3%. Considering that the DS of modified starch with OSA, is influenced by the temperature, pH and reaction time, the results obtained in this study (0.0208) were consistent with those reported by other authors who indicated that the DS in modified starch with 3% of OSA, was between 0.0034 and 0.022 [5,19,25,26].

### 3.2. Effect of the Dual Modification on the Morphology of Corn Starches

**Figure 2:**
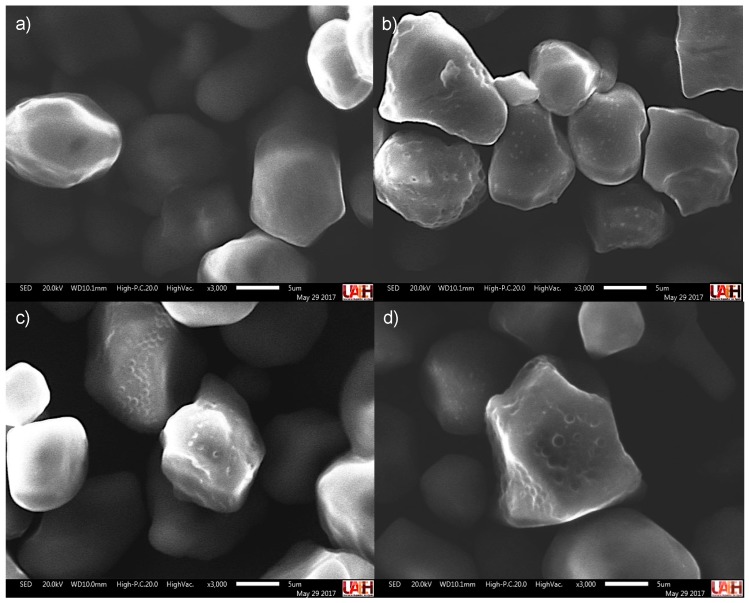
SEM micrographs of corn starch. (**a**) Corn starch without treatment; (**c**) corn starch with acid hydrolysis pretreatment only; (**b**) corn starch with succinylation treatment only; (**d**) corn starch with dual modification.

SEM images of corn starches with or without acid hydrolysis or succinylation are shown in Figure 2. SEM revealed that the exposed surface of the native corn starch was principally smooth with a distinctive polygonal-like shape, similar to the results obtained by Utrilla-Coello et al. [27]. After the native corn starch was subjected to acid hydrolysis pretreatment, some starch granules presented slight exo-erosion that generated a rough surface with indentations or pores. Succinylation resulted in whitish points on the granule surface, which was likely due to the accumulation of succinyl groups, suggesting a compacting of the polysaccharides on the surface of the octenyl succinate-treated starch.

When the native corn starch was subjected to the dual modification, the number of whitish points was lower compared with the starch that was subjected to octenyl succinate without acid hydrolysis pretreatment. This finding indicated a higher degree of incorporation of the succinyl groups into the starch, which was a consequence of the surface erosion, fracture of the granule and polymeric decompaction in the starch that resulted from the acid hydrolysis pretreatment [18]. In the corn starch with dual modification, no wide variations in granule shape and size occurred.

### 3.3. Peak Viscosity (PV)

**Figure 3:**
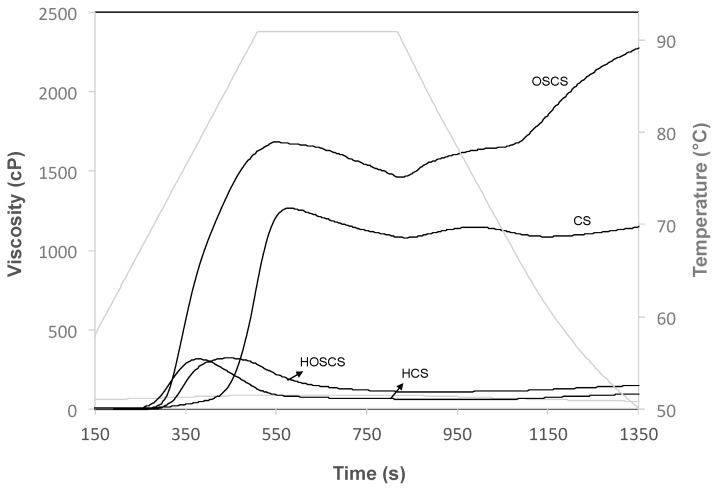
Pasting properties of corn starch. (CS) Corn starch without treatment; (HCS) Corn starch with acid hydrolysis pretreatment only; (OSCS) Corn starch with succinylation only; (HOSCS) Corn starch with dual modification.

The peak viscosity profiles are presented in Figure 3. The acid hydrolysis pretreatment strongly affected the peak viscosity of the starches, which decreased considerably to 1269 ± 1.4 cP and 322 ± 1.4 cP in the starches without succinylation (CS and HCS, respectively). A similar trend was observed for the starch that was treated with octenyl succinate; the viscosity peak values decreased to 1686 ± 4.2 cP and 367 ± 1.4 cP for the octenyl succinate-treated starch without hydrolysis pretreatment (OSCS) and the starch with dual modification (HOSCS), respectively. The low PV of the starch following the acid hydrolysis pretreatment was likely due to an increase in the depolymerization process on the starch, generating weak pastes resulting from hydrolytic effects of the acid modification on the amorphous regions of starch.

The effect of acid hydrolysis on decreasing the PV in starch has been reported by other authors who indicated that the dual modification of acid hydrolysis and citric acid substitution generates a lower PV of the starch extracted from yam cultivars [28].

Acid hydrolysis pretreatment improved the succinylation process, increasing the interaction between the succinyl groups and molecular components of the starch. Previous studies have reported that the pasting properties of starches are modified not only by acid hydrolysis but also by the molecular weight and type of the incorporated ester groups, which potentially promotes cross-linking or molecular networks with amylose or amylopectin [29]. Evidently, dual modification leads to greater damage to the starch components compared with the succinylation process without acid hydrolysis pretreatment. However, some authors have reported that modification with succinyl groups affects the starch structure and consequently, modifies the physicochemical properties of the starch, decreasing the gelatinization temperature and modifying the starch viscosity [30,31].

### 3.4. Differential Scanning Calorimetry Analysis

**Figure 4:**
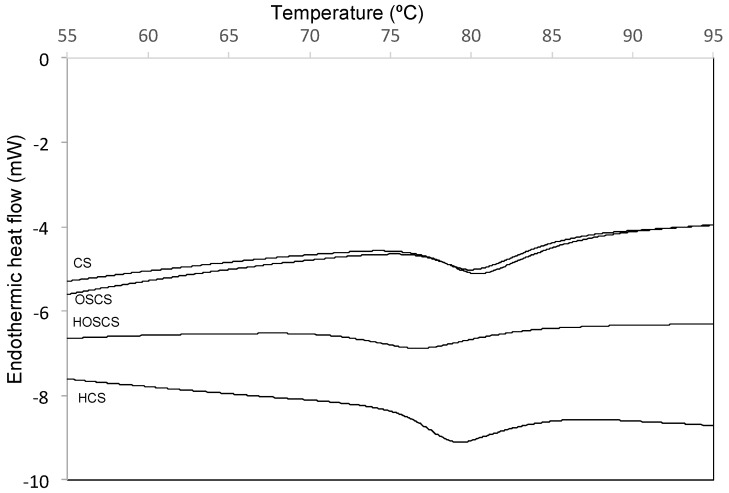
Thermogram of gelatinization of corn starch at 5% (solids), from 35 to 95 °C, at 5 °C/min (CS) Corn starch without treatment; (HCS) Corn starch with acid hydrolysis pretreatment only; (OSCS) Corn starch with succinylation only; (HOSCS) Corn starch with dual modification.

The DSC thermogram is showed in Figure 4. The hydrolyzed corn starch with or without succinylation treatment differed significantly (*p* < 0.05) with respect to native corn starch and octenyl succinate-treated starch without hydrolysis pretreatment. The acid hydrolysis pretreatment of the corn starch influenced the onset temperature (*T*_o_), peak temperature (*T*_p_), concluding temperature (*T*_c_) and the enthalpy for gelatinization (Δ*H*).

As shown in Table 1, the transition temperature of the corn starches with acid hydrolysis pretreatment (HCS and HOSCS) were lower than the transition temperature of the corn starches without acid hydrolysis pretreatment (CS and OSCS). This phenomenon is likely due to the depolymerization process of the starch. On the other hand, the transition temperatures of the octenyl succinate-treated starches (OSCS and HOSCS) were lower than the transition temperature of the corn starch that was not subjected to succinylation (CS and HCS). This property is one of the advantages of succinylation, due to the presence of the succinyl groups [32]. These results suggest that both modification processes (acid hydrolysis pretreatment or succinylation) affect the granule structure, resulting in alterations to the thermal properties of the starch. The modifications to the thermal properties of the modified starches allows for the use of these starches in food processing and reduces the energy required during cooking.

A higher range of gelatinization temperatures (*T*_c_–*T*_o_) was observed in the octenyl succinate-treated starches; this increase indicated that the succinyl groups modified the crystalline structure, interfering with the reassociation of the amylose and amylopectin during the gelatinization process. It has been reported that succinyl groups weaken the hydrogen bonding of the amylopectin, leading to an increase in linear segments that ease the absorption of water into the granule, thereby changing the thermal properties [33,34,35].

It has been reported that any modification or treatment of the starches modify their thermal properties compared with native starches [15,16,36,37]. For this reason, alterations to the thermal properties were more noticeable in the starches that were subjected to the dual modification (acid hydrolysis pretreatment and succinylation). These results are in accordance with those previously published by Betancur-Ancona et al. [32] who reported that the succinylation process modifies the gelatinization temperature of starches, in addition, previous reports have mentioned that the type and nature of the functional group attachment and the degree of esterification have an important effect on Δ*H* [8].

The corn starch with dual modification showed significantly lower *T*o, *T*p, *T*c and Δ*H* than their native counterparts, Sharma et al. [18] and Lv et al. [38] have indicated that the lower gelatinization temperature and enthalpy is due to the reduction of hydrogen bonding by the hydrophobic alkenyl groups, which allows the swelling of the starch at a lower temperature and hence the enthalpy of the corn starch with dual modification decreased. On the other hand, the introduction of voluminous succynil groups into the backbone of the biopolymer enhanced structural flexibility, and contributed to the reduction of gelatinization temperature of the corn starch with dual modification.

### 3.5. FTIR Analysis of Structural Alterations due to the Dual Modification of Starch

**Figure 5:**
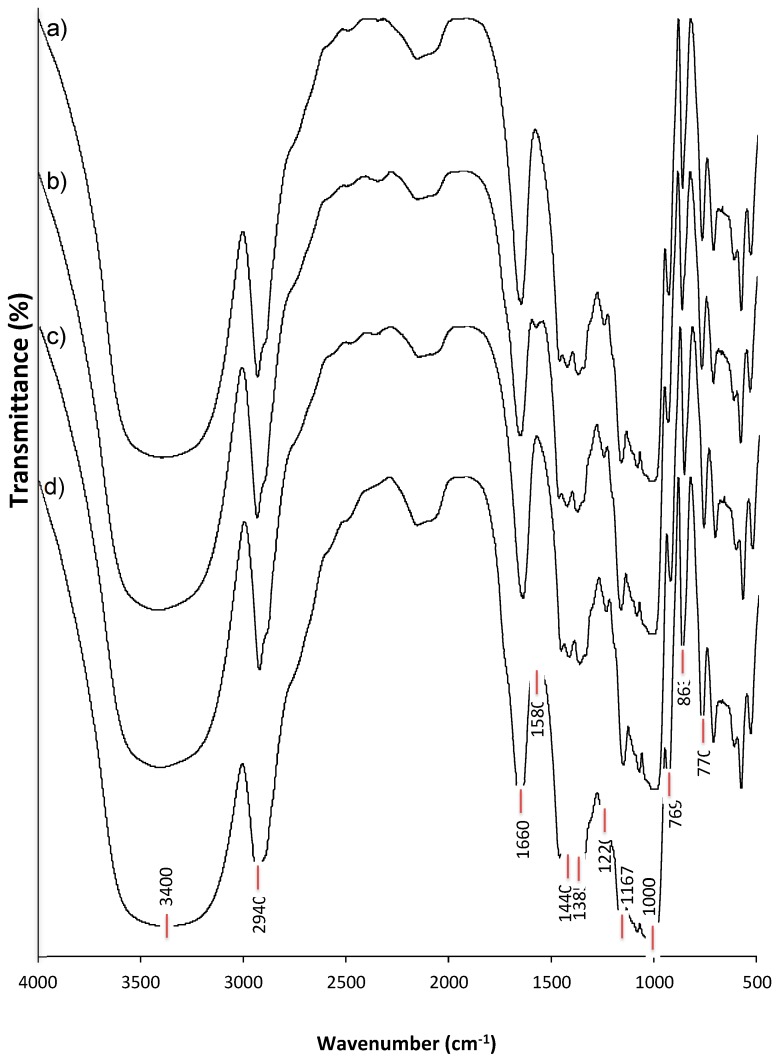
FTIR patterns of corn starch. (**a**) Corn starch without treatment; (**b**) corn starch with succinylation only; (**c**) corn starch with acid hydrolysis pretreatment only; (**d**) corn starch with dual modification.

The FTIR spectra of native corn starch, hydrolyzed corn starch, octenyl succinate-treated corn starch and corn starch subjected to dual modification are shown in Figure 5. Slight modifications can be observed at the wide band centered at 3400 cm^−1^, which corresponds to the stretching vibration of O–H bonds, which is moisture-content dependent. The smoother 3400 cm^−1^ band observed for octenyl succinate-treated starch with or without acid hydrolysis pretreatment suggested the formation of more hydrogen bonds in the starch, which was attributable to the presence of the succinyl groups, which generated more linear segments in the starch and facilitated the absorption of water into the granule [33,34,35].

All starches exhibited a well-defined band around 2940 cm^−1^, which was assigned to the stretching vibration of the C–H bond from the glucose units. A slight modification in the peak intensity of the band around 1660 cm^−1^ was observed only in the starch with the dual modification (Figure 5d), which implies that there were alterations in the crystallinity of the starch. The band around 1660 cm^−1^ was assigned to the scissor vibrations of –OH groups due to the water hydration on the amorphous regions of the starch [39], and the interaction of the starch with the acid and succinic groups apparently involves the hydrolysis of glycosidic bonds and consequently, a loss of amorphous structure due to amylose hydrolysis [40].

The octenyl succinate-treated starches exhibited one new band in the FTIR spectra at around 1573 cm^−1^, which has been related to the asymmetric stretching vibration of carboxylate groups; in addition, in the FTIR spectra of these starches, a slight shoulder appearing at 1730 cm^−1^ was assigned to their carbonyl group. Wang et al. [10] have indicated that the presence of these two new bands (1573 cm^−1^ and 1730 cm^−1^) is indicative that the succinyl group was successfully esterified with the starch. According to Ye et al. [26], Miao et al. [25] and Zhang et al. [41], the intensity of these peaks (1573 cm^−1^ and 1730 cm^−1^) is enhanced when the DS is increased. For this reason, in this study, the signal of these bands in the FTIR spectra of the OSA-starches with an esterification level of 3% was lower than that of OSA-starches with an esterification level of 15%, as reported by Wang et al. [10].

A slight modification was observed in the intensity of the band around 1660 cm^−1^, which corresponds to the scissor vibrations of –OH from hydration water on the amorphous regions of the starch [39]. The dual modification of the starch enhanced the band at 1660 cm^−1^ (Figure 5d), which implies alterations to the starch crystallinity that resulted from amylose hydrolysis due to the interaction of hydrochloric acid-starch-succinic anhydride.

All starches exhibited the characteristic band of the polysaccharides corresponding to the O-H in-plane bending around 1440 cm^−1^; however, there were also two bands around 1460 cm^−1^ and 1380 cm^−1^, which indicated an angular deformation of the C–H in the starch [39,42,43], and a band around 1245 cm^−1^ that is associated with the structural order of starch [42].

The alteration of the crystallinity was confirmed based on the slight change in the ratio of the band intensities of 1047/1022 cm^−1^ (1.28 to 1.22) for the starch without modification (CS) and the starch with the dual modification (HOSCS), respectively; this change indicated a decrease in the crystallinity [44], apparently due to the loss of amorphous structure of the corn starch by effect of the amylose hydrolysis and the interactions of the corn starch with succynil groups.

All starches showed bands around 864 cm^−1^ and 770 cm^−1^, which were related to the stretching vibration of C–O–C and C–O–H from glycosidic bonds. This finding confirmed the α–configuration of the glycosidic linkage in the starch [45]. A well-defined band around 580 cm^−1^ indicated the skeletal modes of the pyranose ring.

### 3.6. Raman Spectroscopy Analysis (FT-Raman) of the Structural Changes of Modified Starches

**Figure 6:**
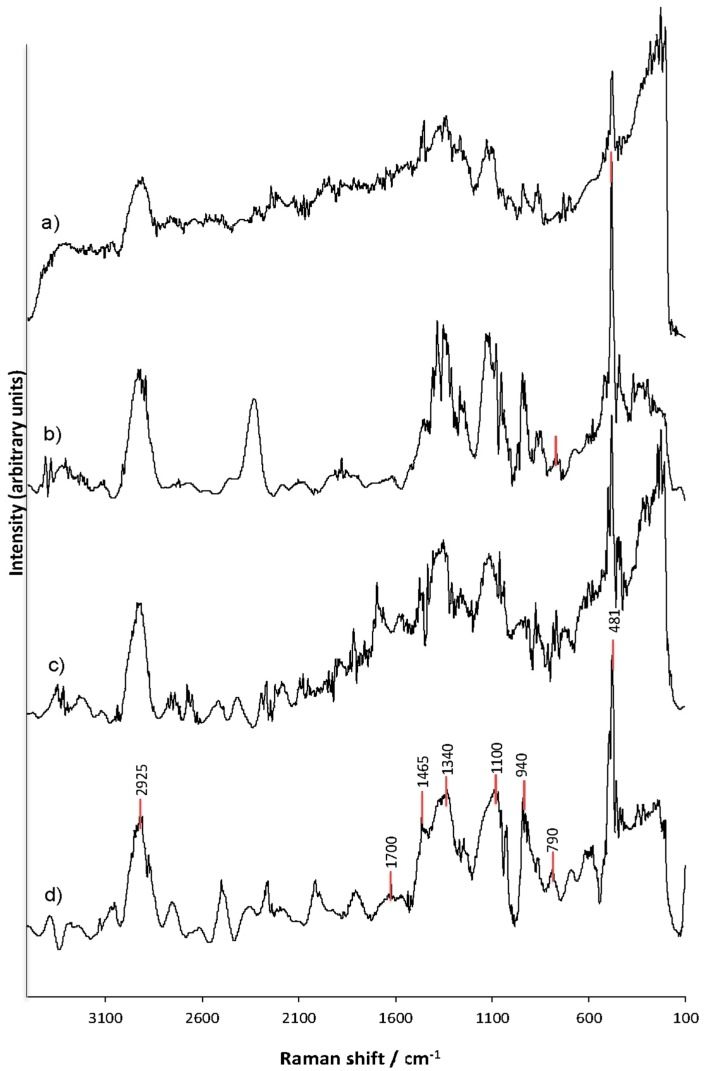
The Raman spectra of corn starches. (**a**) Corn starch without treatment; (**b**) corn starch with succinylation treatment only; (**c**) corn starch with acid hydrolysis pretreatment only; (**d**) corn starch with dual modification.

The Raman spectra of the starch samples are presented in Figure 6. Remarkable differences were observed in the spectra of the starch with dual modification (Figure 6d), while the spectra of corn starch without modifications (Figure 6a) and hydrolyzed corn starch (Figure 6c) were very similar.

The FT-Raman spectra of starches indicated the C-H stretching mode around 2930 cm^−1^, and a change in the intensity of this band was observed in the starch with dual modification (Figure 6d), which could be attributed to variations in the amylose-amylopectin interaction caused by the hydrolysis and succinylation treatments.

The band at around 1700 cm^−1^ due to the C=O stretch of the succinyl functional group [46] was only observed in the starch with the dual modification. Evidently, a rearrangement of the amylose and the amylopectin resulting from the hydrolysis process generated a high succinylation of the starch on the granule surface. According to Wezel et al. [47] the enhancement of this band is clear evidence for the presence of the octenyl succinate ester group at the surface of the modified starch granule. In addition, changes in the Raman spectra resulting from the dual modification of the starches were observed in the region around 940 cm^−1^; this change in the spectra is also evidence for the molecular modification of the starch. These results confirmed that the succinylation of the starch altered the structure of the starch granule and generated an unflattened octenyl succinate-modified starch, as was previously reported by other authors [47].

The most intense band at 481 cm^−1^ that was observed in the starch with dual modification has been reported to be evidence of a disruption of the short-range molecular order in the starch. The effect of this disruption of the short-range molecular order was higher when succinylation is the primary modification (Figure 6b) compared with the acidification pretreatment (Figure 6c). On the other hand, the succinylation process following the acidification pretreatment further increased the disruption of the short-range molecular order; some authors indicate that this effect is because the octenyl succinate derivatization increases the disruption of the short-range molecular order in pretreated corn starch [10].

In general, the bands between 1130 cm^−1^ and 474 cm^−1^ were dominated by C–OH and OH groups, which indicated the intramolecular interactions between polymer chains that are typical in the crystalline regions of starch [48] generated by hydrogen bonds. For this reason, the results of the succinylation-treated starch suggest that the structural changes in the starches with or without hydrolysis pretreatment exhibited an amphiphilic behavior, as the Raman spectra were characterized by modifications in the signals (presence or intensity) between the range of 1130 cm^−1^ and 474 cm^−1^ (Figure 4b,d).

## 4. Conclusions

The dual modification (acid hydrolysis pretreatment and succinylation) of corn starch generated alterations on the surface of the corn starch granule, facilitating the succinylation process and thereby increasing the RE above 50%. The succinylation of the corn starch altered its pasting properties, thereby lowering the PV and Δ*H* and increasing the amphiphilic properties through the rearrangement of the amorphous and crystalline zones. New bands observed in the FT-IR and Raman spectra confirmed that these spectroscopic methods are complementary; further, these bands indicated the presence of succinyl groups in the dual-modified corn starch. This dual modification process may have relevant and suitable applications in the food industry and for the development of materials for the encapsulation of bioactive compounds.

## Figures and Tables

**Table 1:** Thermal properties of corn starch with or without modifications.

Starch Sample	*T*o	*T*p	*T*c	*T*c–*T*o	Δ*H*
CS	76.69 ± 0.18 ^a^	80.45 ± 0.16 ^a^	85.46 ± 0.12 ^a^	8.77	10.94 ± 0.14 ^a^
HCS	65.09 ± 0.28 ^b^	69.45 ± 0.14 ^c^	74.93 ± 0.19 ^b^	9.84	10.57 ± 0.18 ^b^
OSCS	75.91 ± 0.18 ^a^	79.08 ± 0.12 ^b^	84.95 ± 0.16 ^a^	9.04	10.07 ± 0.14 ^b^
HOSCS	60.52 ± 0.27 ^c^	67.09 ± 0.15 ^d^	73.99 ± 0.14 ^c^	13.47	9.14 ± 0.15 ^c^

The means followed by differents letters in the same columns are significantly differents at *p* < 0.05.
